# Pathology and Practice of Medicine

**Published:** 1860-03

**Authors:** Richard J. Dunglison


### PATHOLOGY AND PRACTICE OF MEDICINE.

BY RICHARD J. DUNGLISON, M.D.

I.—*Epilepsy, with Ossification of the Falx Cerebri; History of the Case of Prof. Wm. Pultney Alison, M.D., of the University of Edinburgh.* By PATRICK NEWBIGGING, M.D., etc., Edinburgh.

It is rarely possible, by post-mortem examination of epileptic patients, to detect the true pathology of the disease. Causes occasionally exist which had never been suspected during lifetime, and these, when revealed by dissection after death, throw much light upon obscurity. The case of Prof. Alison is interesting on this account, independently of any importance it may have as the history of the illness and death of a distinguished scientific teacher. In a paper read before the Medico-Chirurgical Society of Edinburgh, by Dr. Patrick Newbigging, the details are given of thirteen years of an existence marked by repeated attacks of a nervous affection, which threatened at any time to place life in jeopardy. While prescribing in the Royal Infirmary, in May, 1846, Prof. Alison fell on the floor in a violent convulsive fit, simulating epilepsy, but to Dr. Paterson, one of the physicians to the hospital, having many of the symptoms of apoplexy. Venesection restored him to consciousness, and soon afterwards to apparently good health. In the same year he had four attacks of a violent nature, and these recurred at intervals of six weeks until 1850, when they became far more numerous. For several years afterwards, their frequency became augmented, and sometimes more violent cerebral symptoms supervened. In October, 1854, he had eleven attacks in the same number of hours, and recovery seemed doubtful. The fits were now far more numerous, so much so, indeed, that it is perhaps rather an under-estimate to say that in the last seven or eight years of suffering, more than three hundred fits occurred.

The last year of his life was distinguished by symptoms of greater danger. Delirium, which accompanied former attacks, now became maniacal, and active restraint was necessary. It was deemed advisable, notwithstanding the cerebral contra-indications, to prescribe large doses of opium instead of the combination of hyoscyamus and camphor, and prussic acid, which had been previously given. Dr. Alison died quietly on the twenty-second of September last, after a violent delirious attack, with intermitting periods of conciousness.

Post-mortem examination, twenty-two hours after death, revealed extensive ossification of the falx cerebri, the anterior portion, to the extent of three inches or more, being converted into a firm bony mass, having a rough, irregular surface, with elevations and depressions which had corresponded with the sulci and convolutions of the brain. A smaller bony mass was detected behind the large ossified portion. The arteries at the base of the brain were also considerably ossified, the pia mater was œdematous, and the surface of the brain, as well as the lateral ventricles, was covered with a serous fluid. The cystic duct was obliterated; no bile was present in the gall-bladder, the organ being filled with large gall-stones, worn smooth by a long-continued process of attrition. No mention is made of the condition of the ductus communis choledochus. No other important pathological appearances were observed, and ossification of the falx was deemed by Dr. Newbigging a very satisfactory means of accounting for the violent symptoms of cerebral disorder manifested during life.

After Dr. Newbigging's report had been presented, a note was read from Dr. James Struthers describing one of the fits. Prof. Alison afterwards made the remark to him, "You will probably be present when my head is examined, and depend upon it you will find no disease of the brain. Whatever disease there may be, you will find it in the membranes; and from what I have observed in similar cases, you will probably find it to consist in a general thickening of those on the upper surface of the brain; but, remember, I have told you there will be no disease of the brain itself." The examination of his encephalon after death verified this prediction. He afterwards, however, expressed the opinion that a tumor must be present in his brain.

The structure of the ossified falx presented characteristics almost analogous to those of true bone, distinct bone-cells, etc., existing. The brain weighed fifty-two ounces, and but for its atrophied condition at the time of post-mortem examination, would, doubtless, have been much heavier.—(*Edinburgh Medical Journal*, January, 1860.)

II.—*Curious Phenomena from the Presence of a Tumor in the Fourth Ventricle of the Brain.* By ALEXANDER WOOD, M.D., President of the Royal Col. of Phys., Edinburgh.

The pathology of the brain, as Dr. Wood remarks, is often our best guide in ascertaining its physiology. It is by the accumulated evidence of symptoms and dissections, that we may occasionally learn much that is interesting in cerebral physiology. In the case recorded by him, post-mortem examination revealed the presence of a tumor lying with its apex in the fourth ventricle, covered on its sides by gray cerebral matter, the remains of the valve of Vieussens, and obstructing the iter e tertio ad quartum ventriculum. The bed of the tumor was formed at the expense of the floor of the fourth ventricle, especially on the right side, and of the corpora quadrigemina, the testes being entirely removed, and the greater part of the nates. It pressed upwards upon the sinuses in that situation, and must have caused some obstruction to the current of blood from the venæ galeni into the straight sinus. The tumor presented all the characters of medullary cancer.

The patient, then a young lady of sixteen, was first seen in 1851, complaining of general debility, but without any marked symptoms of disease. She soon acquired robust health under the use of chalybeate tonics and sea-bathing; her mental powers and vivacity were developed beyond her years, and her imaginative faculty was extraordinarily quick. Two years afterwards, it was noticed that her fondness for reading and the exercise of her intellect had given place to carelessness of her studies. In 1855, she suffered from amenorrhœa and general debility, the extent of which could scarcely be accounted for from the constitutional symptoms. In 1856, her sight began to be impaired, and she had not full power in directing the movements of her limbs. During all this time, and, indeed, through all her illness, she presented the picture of perfect health; the dimness of vision she ascribed to the reading of small type; the weakness of the limbs Dr. Wood hoped might be accounted for by the menstrual suppression. In March of that year, the symptoms became greatly intensified—there was decided dragging of the right leg, the eyes seemed prominent and staring, and rolled as if she had not the power of controlling their movements. Cerebral mischief, before suspected, was now positively diagnosticated by her physician.

In the summer of 1856, she was unable to walk unsupported; the pupils were dilated, and soon afterward she was able only to discern light from darkness. She suffered from occasional convulsions; the intestinal canal became totally inactive, rarely responding to the action of powerful purgatives, and she was unable from this time, the summer of 1856, up to the time of her death, to pass a single drop of urine unless the excito-motory system was stimulated. The difficulty appeared to depend partly on the absence of sensation, or of the centripetal nervous influence in the bladder itself, and partly on paralysis of the abdominal muscles. Mental excitants, such as a fit of laughing, caused the urine to flow freely, and a like effect was produced by the dashing of water on the abdomen. Her mind continued active, and her memory became extraordinarily developed.

After several weeks of mitigation of the prominent symptoms, she had decided prodromic evidences of meningitis, in December, 1856, as well as in July, 1857, both of which attacks were averted, however, by remedial agents. In October, 1857, she complained of noises in her head, like the ringing of bells or the rushing of a torrent, and visions of balls of fire before her eyes. Yet her intellect was still astonishingly clear. A curious peculiarity was that she had, regularly on alternate days, her dark day and her light day—seeing black objects like coffins on the former, and flashes and balls of fire on the latter day. Her eyes would not, to a casual observer, indicate blindness. Her arms became paralyzed, and useless to her, and she lost flesh rapidly. As her physical sufferings became greater, her intellect and memory seemed to acquire wonderful strength. From the 3d of January, 1858, to the 8th, (the day of her death,) she suffered from excessive vomiting and intense headache, the pain recurring on one or two evenings at hourly intervals. On the eighth of January, the paralysis extended to the pharynx, and in the evening the muscles of the left side of her face became violently convulsed for nearly an hour. Her face had the appearance of perfect health, her intellect was quite clear, she directed the attendants where to place the local applications to her head, and had just asked that her lips might be wiped, when she died.

In this remarkable case, it is not a matter of surprise that general paralysis should have occurred, when we know how deeply the thalamus opticus was found to be implicated by the tumor. The pressure on the optic tubercles would account for the amaurosis, while the position of the fourth nerve, as regards the tumor, might account for the curious rotation of the eyeball. Perhaps the most remarkable point in the history of the case was the uninterrupted continuance of intellectual vigor, and increase of memory, and the absence of stupor until immediately before death, while we may find as much difficulty in explaining the curious regular alternations of vision—"the photopsic and chromopsic appearances" which Dr. Wood has well described. It may be remarked, in regard to the difficulty of micturition, from which she so long suffered, that strychnia, nux vomica, and the other so-called reflex stimuli, were totally inefficacious in restoring the power of emptying the bladder.—(*Edin. Med. Jour.*, Jan., 1860.)

III.—*Effect of Cerebellar Hemorrhage on Muscular Motions.* By M. HILLAIRET.

The functions of the cerebellum have long been studied by physiologists, and many experiments made by vivisection to determine how any lesion of that part of the cerebro-spinal axis might affect the movements or sensations of the animal. The theory that this important nervous organ presides over motion receives confirmatory evidence from a case described to the French Academy of Sciences by M. Hillairet. A man of very advanced life was seized with an apoplectic attack, attended by no loss of intelligence during its continuance. He had severe cephalalgia on the right side, and it was upon the right side that he fell at the commencement of the attack. He lay upon this side also, a sort of rotation of the body occurring, and every attempt to move him occasioned vomiting or nausea. After the disappearance of some of these symptoms, it was found that attempts to rise or stand up occasioned a fall forward or to the right side, while, at every effort at motion forward, his legs took a different course from that intended. Cerebellar hemorrhage was diagnosticated, and M. Hillairet had the satisfaction, several months afterwards, when the patient died of the effects of a cerebral hemorrhage, to find the trace of a previous hemorrhage in the centre of the white substance of the right hemisphere of the cerebellum, as well as a more recent clot on the left thalamus opticus. The latter had produced hemiplegia of the opposite or right side of the body.

The details of this case seem to favor the views of M. Flourens and other physiological inquirers, that the cerebellum is the great regulator of locomotive movements, and also bear out the assertions of other observers, that injury to the cerebellum produces a recoiling movement, independently of every effort which the will is making to a forward direction.—(*London Lancet*, Dec. 3, 1859.)

IV.—*Cerebral Symptoms Independent of Cerebral Disease.* By CHARLES WEST, M.D.

In a lecture delivered at the Hospital for Sick Children in London, Dr. West made some general remarks upon the importance of distinguishing between diseases of children depending on cerebral causes, and those which presented merely cerebral symptoms. "Do you think his brain is affected?" is a question frequently asked, and the physician's reply must be governed by his knowledge of the distinctions between true cerebral disorder and that which similates it. Heaviness, drowsiness, a feeling of unrest, are precursory symptoms of so many affections, that it becomes necessary to detect, if possible, their real import. The younger the child, of course the more difficult will be the diagnosis. Dr. West considers three or four important points:—

1st. "The cerebral symptoms that usher in the attack, or accompany the early stages of fever." The more violent these are at first, the less likely are they to be dependent on disease of the brain; violent convulsions, for example, at this stage, being almost always connected with some morbid condition existing elsewhere. So, too, in the early stages of eruptive fevers, as measles and small-pox, convulsions of a violent nature occur, but disappear on the decided appearance of the eruption. When, however, very severe convulsions precede an attack of epidemic scarlatina, the prognosis may generally be formed of a malignant type of the disease. At a time when epidemic scarlet fever visited Dresden, in 1831 and 1832, cases were seen of intense cerebral disorder without any eruption, differing from inflammation of the brain in the rapidity of attack and in the absence of many of the symptoms, and yet, by post-mortem examination, the conclusion was arrived at that these were cases of scarlatina, in which death had taken place before time was given for the manifestations of the disease. Sometimes a question arises whether the affection of the little sufferer is tubercular hydrocephalus or typhoid fever; but the character of the cerebral symptoms is different, and age also may often determine the diagnosis,—the younger the child the less likely the disease to be hydrocephalus.

2d. "Those [symptoms] which at the outset of acute inflammations of the thoracic viscera sometimes throw the evidences of the real disease into the shade." Pneumonia and pleurisy, for instance, occasionally commence with a convulsion and vomiting, and delirium may follow, while the cough may be slight. Looking at the preponderance of severe cerebral symptoms over the evidences of thoracic disorder, cerebral congestion, hydrocephalus, or typhoid fever may be alike erroneously diagnosticated. But the physician cannot long remain in error, especially if he has the diagnostic skill to recognize the absence of many of the symptoms of diseased brain.

3d. "Those [symptoms] dependent on some disorder of the abdominal viscera," etc. Convulsions from such causes depend often on the indigestible character of the food supplied to the child, but the history of the case, the condition of the abdomen, and the peculiar character of the convulsion will generally establish the diagnosis. In diarrhœas, and relapses of diarrhœas, a condition may supervene, where the case has been persistent, in which irritation and exhaustion may produce symptoms like those of inflammation of the brain—hydrocephaloid disease, a kind of spurious hydrocephalus. The same condition may arise from the gradual influence of imperfect nutrition, the cerebral symptoms coming on slowly and insidiously. As fatal results may follow this imitative disease as attend true inflammation of the brain, and yet if a treatment be adopted which aims at correcting the cause, instead of one founded upon a faulty diagnosis, such results may be averted.

4th. "Chronic cases, in which cerebral symptoms occur and are likely to be misapprehended." Dr. West briefly sketches a few of these, such as temporary but long-continued dulling of the faculties after fevers, altered temper, a precociousness which becomes alarming from its unexpectedness, neuralgic headache, so often thought to be the precursor of mental disease in children, etc.

In these warnings against the errors which may so readily occur in a hurried diagnosis, Dr. West has given valuable hints and suggestions of dangers which may attend the practice of physicians of any age in their care and management of the young sick.—(*Medical Times and Gazette*, December, 1859.)

### V.—*Fatty Degeneration and Acute Softening of the Liver, with Entorrhagia.* By M. L. MARCQ.

M. Marcq presented to the *Société Anatomo-Pathologique de Bruxelles,* in May last, a pathological specimen of diseased liver, on which he made some practical remarks. A young girl, apparently in perfect health, had suddenly been attacked with rigor and nausea, for which she could assign no cause. She soon after passed by the bowels a small quantity of blood. This condition of nausea and general indisposition lasted for several days, at the end of which time she was brought to the Hôpital Saint-Pierre, with intense headache; her abdomen was tympanitic, her hepatic region very painful. When Professor Uytterhoeven first saw her, she presented the appearance of a parturient woman at the commencement of puerperal intoxication, lying on her back, her limbs completely relaxed, her countenance expressing painful and profound prostration. Her general hue was that jaundiced tint by which malarious impression so often leaves its mark. She was conscious, although roused with difficulty to the proper attention. In the evening of the same day, the skin was cold, the pulse frequent but small, her desire to eat very great, and every evidence existing that the case was a rare form of affection, to which the attention of the hospital had not previously been directed. Professor Uytterhoeven had seen a similar case at the Hôpital St. Jean, and from his recollection of the pathological appearances then revealed, diagnosticated this to be a form of acute softening of the liver.

The treatment adopted was more expectant than active. The sulphate of quinia, which at first suggested itself, was deemed too irritating in the existing condition of the alimentary passages. The next night the patient died, four days after the appearance of the first symptoms. On a post-mortem examination, thirty-six hours after death, the liver was found to be the only organ seriously affected. Its surface was adherent to the diaphragm, and covered with patches, the results of inflammation, which extended into the substance of the organ. The liver was intensely yellow, softened, and diffluent under pressure of the finger. Microscopic examination detected numerous fat globules swimming in an almost colorless liquid, and but few traces of the true substance of the organ. Nearly a quart of black, grumous blood was found in the stomach and intestines. In this case no atrophy of the liver occurred, although existing in the cases recorded up to that time. M. Marcq, therefore, prefers the name which forms the heading of this abstract to that of *acute atrophy*, which had previously been given to the disease.

English and German writers were the first to describe the pathology of this comparatively rare affection. The views of Dr. Fried. Frerichs are cited in regard to the symptomatology, pathological anatomy, and treatment of the affection; but the general summary presents, in different degrees of intensity, the same general symptoms as those described above, while the treatment seems to be founded more upon general principles than upon any specific modes of relief. —(*Annales de la Société Anatomo-Pathologique de Bruxelles, Bulletin*, No. 2, 1859.)

VI.—*Chylous or Milky Urine.* By C. E. ISAACS, M.D.

The Transactions of the New York Academy of Medicine, vol. ii. part iv., contain interesting remarks upon a rare form of disease, whose pathology is not definitely settled. Two illustrations of the affection occurred at the Seamen's Retreat, Staten Island, under the care of Dr. T. C. Moffatt, chief physician of that institution. The first case was a Spaniard, who was admitted November 13th, 1858, for abscess of the scrotum. He had noticed three years previously that his urine was occasionally of the color of milk, and remained so for several years. It is now noticed that the urine voided after meals is always milky. When retention sometimes occurs for a few hours, the ejection of a long, fibrinous coagulum precedes the flow of milky urine. He had had gonorrhœa and syphilis, and had taken large quantities of mercury and iodide of potassium. Long-continued pain in the lumbar region preceded the first apparent change of color in the urine. The early morning urine is clear, but becomes milky after breakfast.

When subjected to examination, it was found to coagulate by heat, nitric acid, and alcohol. That the milk-white color of the urine was due to an intimate mixture of oily matter with albumen, forming a kind of emulsion, was proved by agitating a small quantity of the urine with sulphuric ether. The urine instantly separated into two distinct portions; the upper layer, of a deep-yellow color, being composed of the ether; while the inferior layer, brownish-yellow and semi-transparent, consisted of albumen. The upper layer left, on evaporation, a copious deposit of oil. No red globules or tube-casts were discovered, after very careful examination. To sum up the various results of chemical tests and microscopical examinations, it was conclusively exhibited that the principal normal constituents of the urine, probably all of them, were present. The same results were obtained from different specimens. He took gallic acid, ℈j to ʒss, three times a day, but was sent home before any beneficial result that might follow could be studied.

The second case, a native of Santa Cruz, a sailor, who had been occupied for five days in taking in a cargo of spirits of turpentine, was admitted in 1855 for hæmaturia, and was soon after discharged cured. In a subsequent voyage, while at Sidney, N. S. W., he was under treatment for periosteal swelling of the tibia, and ten weeks before sailing he experienced constant desire to urinate, and noticed that small clots of blood at times interrupted the free flow of urine. Soon after sailing, he observed that his urine was milky, and he was obliged to urinate about twenty times in the twenty-four hours. The appearances in this case differed somewhat from those observed in the other. The urine became like solidified jelly five minutes after it was voided; fibrinous lumps and strings passed with the fluid—some of them yellow, others reddish—varying in size frôm a pea to a large pigeon's egg; all contained myriads of red globules. When the urine was allowed to stand, a red layer formed at the bottom of the vessel. The odor of the urine was, in both cases, that of moist clay. No oil globules were detected under the microscope in the fluid portion that remained after the separation of the coagulum. Nitric acid produced coagulation, but this result was not attained by the application of heat until several weeks afterwards. He was put upon the use of gallic acid, and afterwards upon tannin and alum. In October, 1858, he passed a gallon a day; and this quantity was not influenced by diet or exercise. Gradually, under the use of good diet, the milky appearance disappeared and the coagulum became very slight; but he finally passed into a typhous condition, which terminated in death early in 1859.

Post-mortem examination of the kidneys showed them to be in a healthy condition.

In a case reported in the *Medical Times and Gazette*, by Dr. Bence Jones, the patient had passed milky urine for twenty-five years. Dr. Golding Bird relates the case of a woman who passed milky urine on rising from bed. Other cases are reported by different writers. The cause is, probably, exposure to external influences, and in the second case mentioned by Dr. Isaacs, perhaps inhalation of spirits of turpentine, while, so far as has been observed, it is a disease of hot climates. Dr. Isaacs arrives at several conclusions, founded on recorded cases and opinions:—1. That the disease may continue for months and years without much apparent injury to the general health. 2. That there may be intermissions of healthy conditions of the urine, perhaps in the same day. 3. That there may be little or no emaciation, and the patient may abound in adipose tissue. 4. That, generally, the fatty matter appears in the urine after eating.

5. That astringents, and especially gallic acid, seem beneficial, but that the disease is very rarely under the permanent control of medicine. 6. That in two cases examined, the kidneys were healthy. 7. That there were probably no organic lesions of any other organs.

We coincide with Dr. Isaacs in the remark that the number of cases recorded has been so small that the true pathology cannot yet be readily determined.—(*Transactions of the N. Y. Academy of Medicine*, vol. ii. part iv., 1859.)

